# Parathyroidectomy in the treatment of BMD-DRC Brazil: many limitations, but still fundamental

**DOI:** 10.1590/2175-8239-JBN-2018-0180

**Published:** 2018-11-14

**Authors:** Lilian P. F. Carmo

**Affiliations:** 1 Universidade Federal de Minas Gerais, Faculdade de Medicina, Belo Horizonte, MG, Brasil.; 2 Centro de Nefrologia do Hospital Evangélico de Belo Horizonte, Belo Horizonte, MG, Brasil.

Secondary hyperparathyroidism (HPS) is a common and serious complication in the course of chronic kidney disease (CKD), with a direct impact on the morbidity and mortality of these patients.^1^ Despite advances in the clinical treatment of HPS with the use of vitamin D analogues and calcimimetics, treatment failure still occurs in a significant number of patients.^1^^,^ 2

In patients with persistently elevated parathormone (PTH) levels (> 800 pg/mL for > 6 months) associated with resistance to clinical treatment, a monoclonal proliferation with nodular hyperplasia is probably present with reduced expression of calcium and of vitamin D. In such cases, parathyroidectomy (PTx) should be considered, especially if it is associated with hypercalcemia, hyperphosphatemia or vascular or tissue calcification.^3^

PTx is required in 15% of patients after 10 years, and in 38% of patients after 20 years of hemodialysis. It is associated with a 15-57% improvement in the survival of patients on hemodialysis and improvement of hypercalcemia, hyperphosphatemia, tissue calcification, bone density and quality of life.^3^

This procedure is in decline in the world. American data show that between the 1990s and the early 2000s, PTx rates reached 10/1000 patient-year. Since then, there has been a progressive reduction in PTx rates, reaching a nadir of approximately 3.3 per 1000 patient-year on dialysis in 2004.^4^ Nevertheless, there was an increase in the mean PTH levels, probably reflecting the introduction of the use of calcimimetics and the uncertainty regarding ideal levels of PTH.^3^

PTx is a challenging procedure that requires great skill and experience from surgeons. Currently, there are adjuvant techniques performed intraoperatively, such as cryopreservation, neurophysiological monitoring, PTH dosing and imaging with sestamibi, which aims to improve surgical outcomes. However, these methods are still largely unavailable, especially in developing countries. The study by Neves et al., published in this issue, evaluates the results of five years of a reference center with a high number of procedures, without adjuvant techniques. They reported a low rate of complication and failure. An important fact described in the text is that a single surgeon, which undoubtedly reflects a unique experience with impact on good outcomes, even in the absence of adjuvant techniques, performed all procedures.

The data from this study show a low sensitivity of the PTx preoperative imaging tests for the location of ectopic and supernumerary glands. In fact, the literature is controversial at this point, especially in relation to surgical outcomes.^5^ The gold standard is still the exploration and location of the glands by the surgeon during the surgery. This data highlights the need to review the recommendations for preoperative examinations (which situations would be necessary and which combination of tests), especially if we take into account the low availability in most municipalities, especially in the public healthcare network, besides being a dependent operator. Above all, it is important to emphasize that the unavailability of imaging tests for the preoperative location of the glands should not prevent or contraindicate surgery.^6^

In this series, the majority of PTx was performed using the total parathyroidectomy technique with autoimplantation. The literature does not show significant differences in surgical outcomes between the different techniques (total with implant and subtotal);^3^ however, subtotal PTx carries a lower risk of permanent hypoparathyroidism in the postoperative period. The effects of low levels of PTH are not clear, but there is always a concern of turning a high remodeling disease into low remodeling, with its potential adverse effects in relation to vascular calcification and lack of therapeutic possibilities in this context. Therefore, the evaluation of the impact of persistent hypoparathyroidism should ideally be performed to define the choice of the best surgical technique in each center.

The publication of the Clinical Protocol and Therapeutic Guidelines for Mineral and Bone Disorders by the Ministry of Health in 2017 included a greater number of treatment options, such as calcimimetics.^7^ The recent increased availability of these new drugs is expected to reduce the number of PTx in the next years, as already reported in other countries after the start of the use of calcimimetics. However, there is currently a significant number of patients considered refractory to clinical treatment. In addition, despite great advances with the new protocol, the medication is only released for patients on hemodialysis or peritoneal dialysis, not contemplating renal transplants, and only for patients with severe hyperparathyroidism, who are highly likely to be refractory to clinical treatment. Thus, PTx remains an essential treatment option for HPS in Brazil. The study by Neves et al. demonstrated that in centers with specialized teams, this procedure could be performed safely, with low complication rates and high success rates, even without the adjuvant techniques.^8^
